# Supplementary material for: Rapid changes in plasma corticosterone and medial amygdala transcriptome profiles during social status change reveal molecular pathways associated with a major life history transition in mouse dominance hierarchies
Source: PLoS Genet. 2025 Jan 13;21(1):e1011548. doi: 10.1371/journal.pgen.1011548 (PMC11761145; doi:10.1371/journal.pgen.1011548)

**Supplemental Figure 1:** A) Experimental design. Each procedure was delayed by a day for half the cohorts that went through a social reorganization at 70 minutes to ensure the same start time for all social reorganizations. GD = Group House Day B) Mice are housed in a 4 per cage system (cage lids not shown).


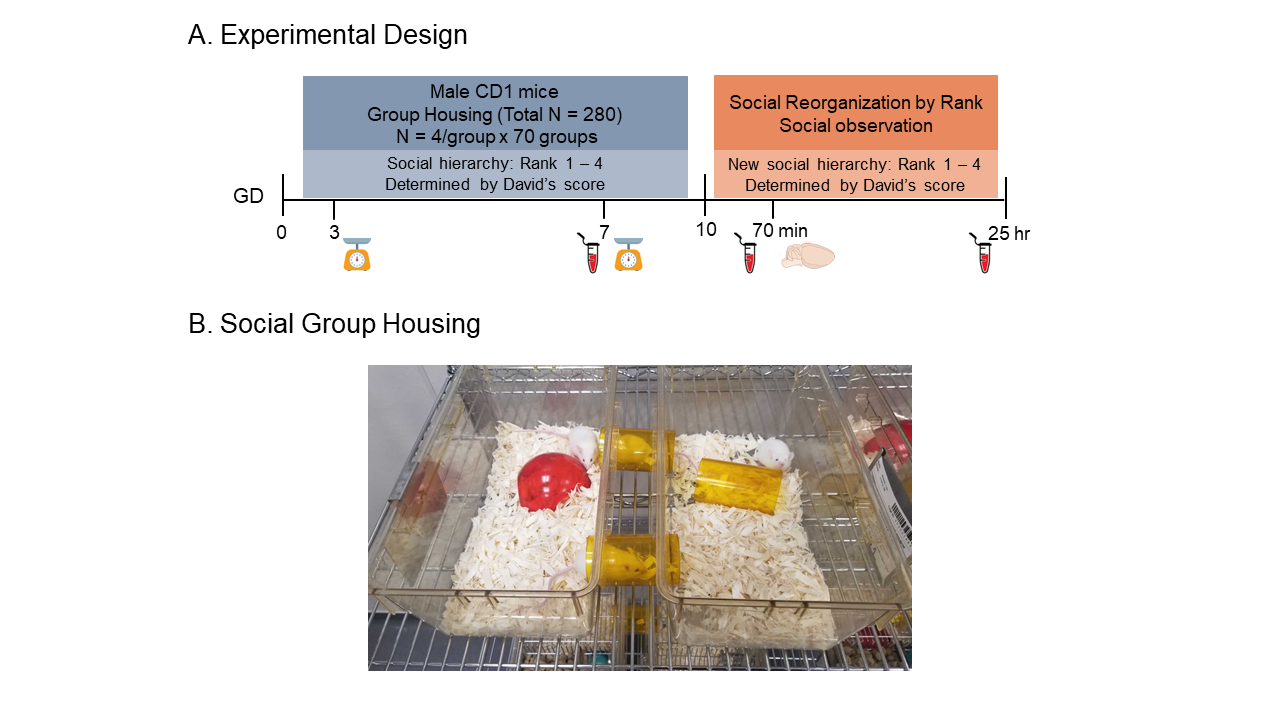

Supplement: S1 Fig — A) Experimental design. Each procedure was delayed by a day for half the cohorts that went through a social reorganization at 70 minutes to ensure the same start time for all social reorganizations. GD = Group House Day B) Mice are housed in a 4 per cage system (cage lids not shown). Brain: https://www.clker.com/clipart-mouse-brain.html Weighing scale: https://creazilla.com/media/clipart/842748/weighing-scale The Eppendorf: https://www.clker.com/clipart-1ml-eppendorf-tube-16.html (DOCX) [file pgen.1011548.s002.docx]
