# Supplementary material for: Rapid changes in plasma corticosterone and medial amygdala transcriptome profiles during social status change reveal molecular pathways associated with a major life history transition in mouse dominance hierarchies
Source: PLoS Genet. 2025 Jan 13;21(1):e1011548. doi: 10.1371/journal.pgen.1011548 (PMC11761145; doi:10.1371/journal.pgen.1011548)
Supplement: S2 Fig — A) Raw Pre-Reorganized sociomatrices of wins and losses for each cohort. Each value represents the total number of wins by the individual in each row against the individual in each column. The degree of redness represents the frequency of wins. B) Binary matrices for each cohort. A 1 in a cell represents that the individual in the row is a consistent winner against the individual in the column. Numbers on rows and columns refer to animal IDs. C) Raw Post-Reorganized sociomatrices of wins and losses for each cohort. Each value represents the total number of wins by the individual in each row against the individual in each column. The degree of redness represents the frequency of wins. D) Binary matrices for each cohort. A 1 in a cell represents that the individual in the row is a consistent winner against the individual in the column. Numbers on rows and columns refer to animal IDs. (DOCX) [file pgen.1011548.s003.docx]

**Supplemental Figure 2:** A) Raw Pre-Reorganized sociomatrices of wins and losses for each cohort. Each value represents the total number of wins by the individual in each row against the individual in each column. The degree of redness represents the frequency of wins. B) Binary matrices for each cohort. A 1 in a cell represents that the individual in the row is a consistent winner against the individual in the column. Numbers on rows and columns refer to animal IDs.


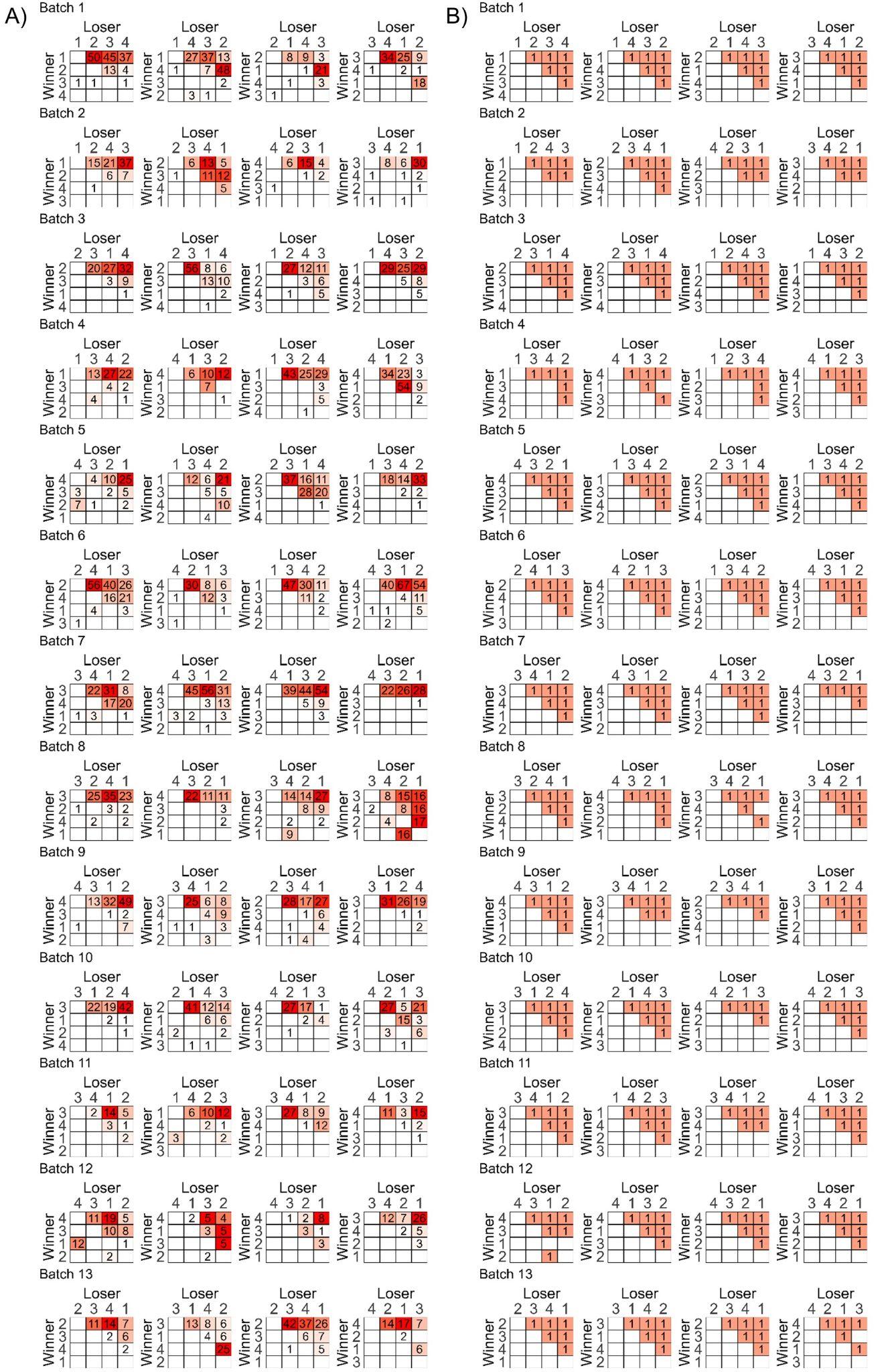


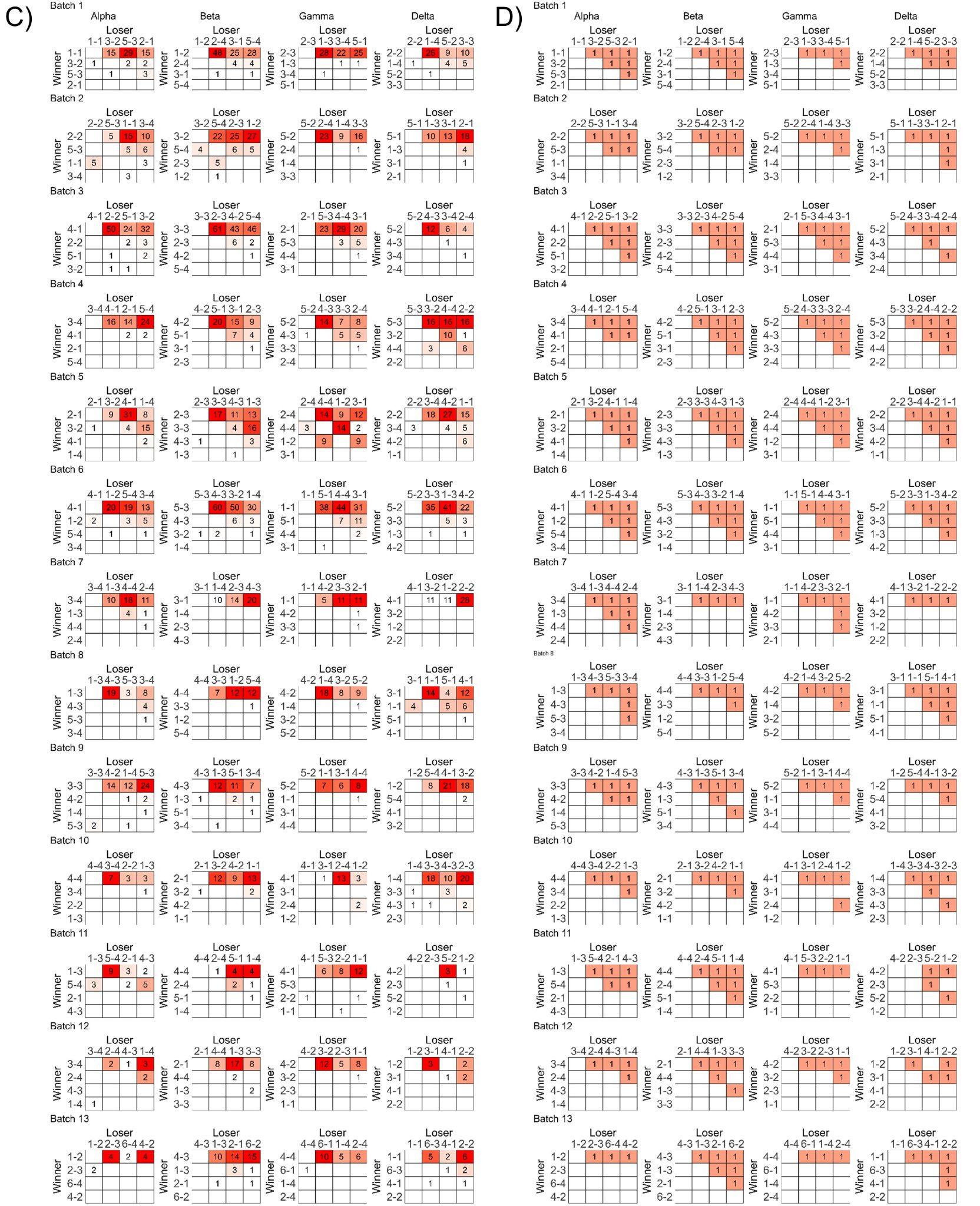
**Supplemental Figure 2:** C) Raw Post-Reorganized sociomatrices of wins and losses for each cohort. Each value represents the total number of wins by the individual in each row against the individual in each column. The degree of redness represents the frequency of wins. D) Binary matrices for each cohort. A 1 in a cell represents that the individual in the row is a consistent winner against the individual in the column. Numbers on rows and columns refer to animal IDs.
