# Supplementary material for: Rapid changes in plasma corticosterone and medial amygdala transcriptome profiles during social status change reveal molecular pathways associated with a major life history transition in mouse dominance hierarchies
Source: PLoS Genet. 2025 Jan 13;21(1):e1011548. doi: 10.1371/journal.pgen.1011548 (PMC11761145; doi:10.1371/journal.pgen.1011548)

**Supplemental Figure 4:** Boxplots showing median and IQR of body mass by pre-reorganization social rank at the beginning of social observation on day 3 or 4 of group housing (A) and once groups were stable on day 7 or 8 (B) of group housing. Points represent individuals.


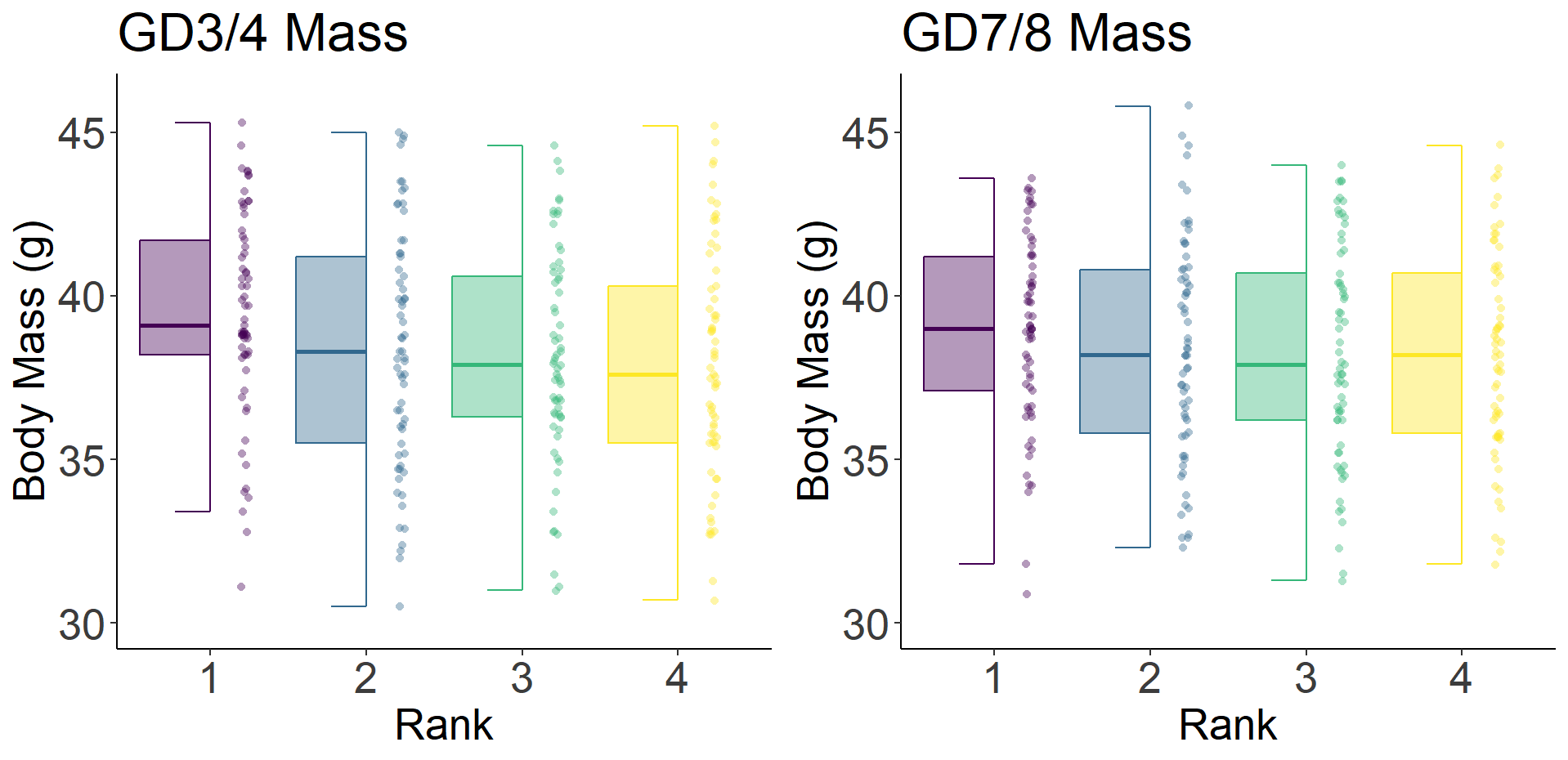

Supplement: S4 Fig — Boxplots showing median and IQR of body mass by pre-reorganization social rank at the beginning of social observation on day 3 or 4 of group housing (A) and once groups were stable on day 7 or 8 (B) of group housing. Points represent individuals. (DOCX) [file pgen.1011548.s005.docx]
