# Supplementary material for: Rapid changes in plasma corticosterone and medial amygdala transcriptome profiles during social status change reveal molecular pathways associated with a major life history transition in mouse dominance hierarchies
Source: PLoS Genet. 2025 Jan 13;21(1):e1011548. doi: 10.1371/journal.pgen.1011548 (PMC11761145; doi:10.1371/journal.pgen.1011548)

**Supplemental Figure 5**: Total frequency of fights in each group 70 minutes after social reorganization. Boxplots represent medians and IQRs. Points represent individuals.


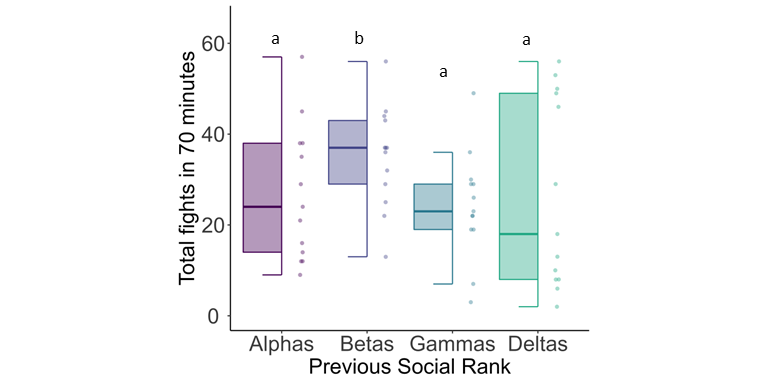

Supplement: S5 Fig — Boxplots represent medians and IQRs. Points represent individuals. (DOCX) [file pgen.1011548.s006.docx]
