# Supplementary material for: Rapid changes in plasma corticosterone and medial amygdala transcriptome profiles during social status change reveal molecular pathways associated with a major life history transition in mouse dominance hierarchies
Source: PLoS Genet. 2025 Jan 13;21(1):e1011548. doi: 10.1371/journal.pgen.1011548 (PMC11761145; doi:10.1371/journal.pgen.1011548)

**Supplemental Figure 6**: Correlation of Rates of A) aggression given or received prior to social reorganization (x-axis), or B) total aggression in each group (x-axis) and post-reorganization David’s score (y-axis). Each facet represents individuals in post-reorganization groups of 1) previously alpha males, 2) previously beta males, 3) previously gamma males, 4) previously delta males. Higher rates of aggression in pre-reorganization groups are associated with higher dominance scores post-reorganization in previously beta males. C) Group of Origin leads to similar post ranks. The green distribution is the theoretical distribution of differences in post-reorganization ranks between animals from the same pre-reorganization groups. The dashed line represents the observed differences in post-reorganization ranks.


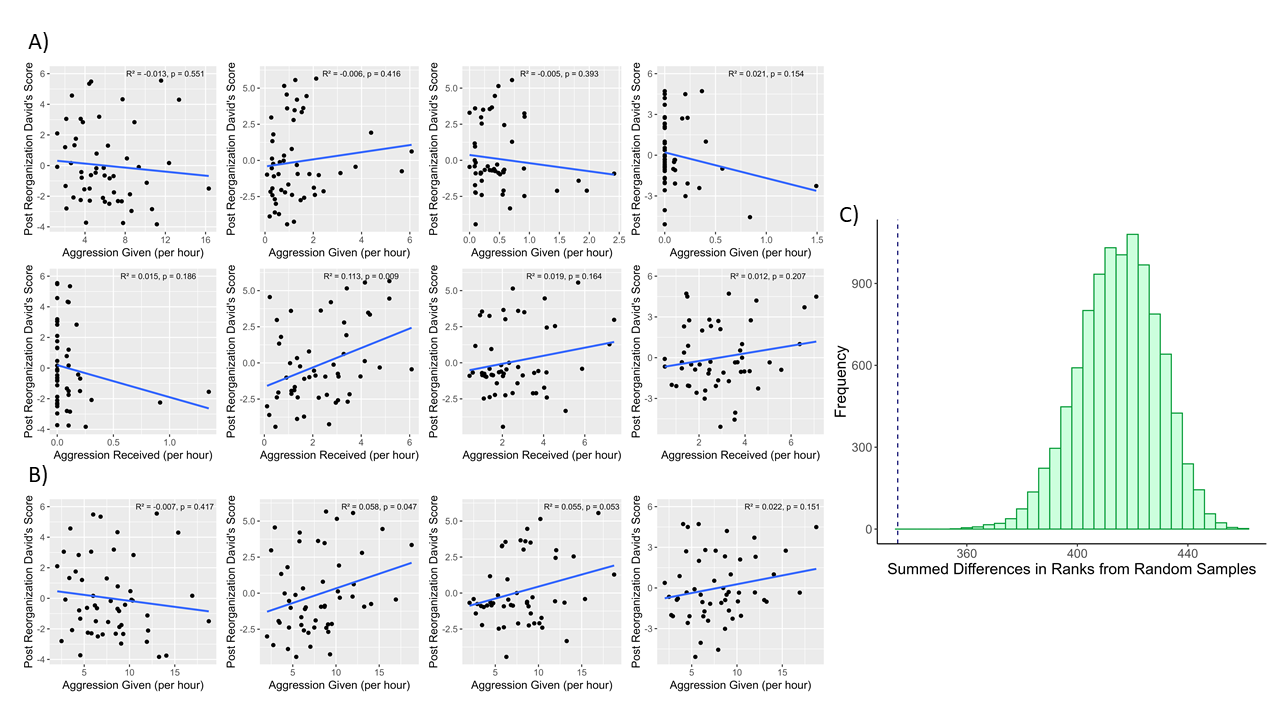

Supplement: S6 Fig — Correlation of Rates of A) aggression given or received prior to social reorganization (x-axis), or (B) total aggression in each group (x-axis) and post-reorganization David’s score (y-axis). Each facet represents individuals in post-reorganization groups of 1) previously alpha males, 2) previously beta males, 3) previously gamma males, 4) previously delta males. Higher rates of aggression in pre-reorganization groups are associated with higher dominance scores post-reorganization in previously beta males. C) Group of Origin leads to similar post ranks. The green distribution is the theoretical distribution of differences in post-reorganization ranks between animals from the same pre-reorganization groups. The dashed line represents the observed differences in post-reorganization ranks. (DOCX) [file pgen.1011548.s007.docx]
