# Supplementary material for: Rapid changes in plasma corticosterone and medial amygdala transcriptome profiles during social status change reveal molecular pathways associated with a major life history transition in mouse dominance hierarchies
Source: PLoS Genet. 2025 Jan 13;21(1):e1011548. doi: 10.1371/journal.pgen.1011548 (PMC11761145; doi:10.1371/journal.pgen.1011548)

**Supplemental Figure 7:**  No significant differences between (A) control and reorganized males or (B) dominant and subordinate males in plasma corticosterone prior to social reorganization. Boxplots represent medians and IQRs. Points represent individual subjects.


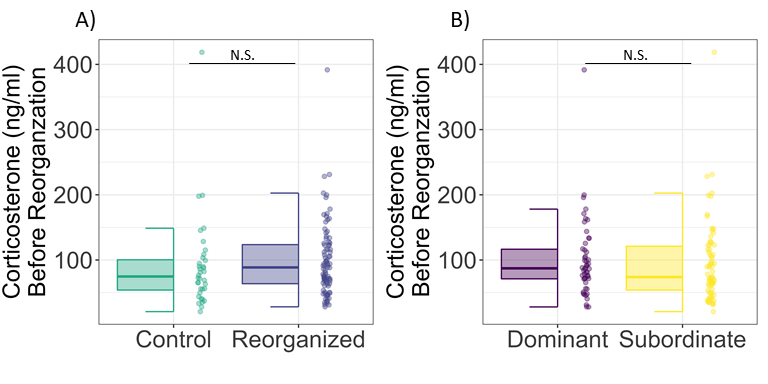

Supplement: S7 Fig — No significant differences between (A) control and reorganized males or (B) dominant and subordinate males in plasma corticosterone prior to social reorganization. Boxplots represent medians and IQRs. Points represent individual subjects. (DOCX) [file pgen.1011548.s008.docx]
