# Supplementary material for: Rapid changes in plasma corticosterone and medial amygdala transcriptome profiles during social status change reveal molecular pathways associated with a major life history transition in mouse dominance hierarchies
Source: PLoS Genet. 2025 Jan 13;21(1):e1011548. doi: 10.1371/journal.pgen.1011548 (PMC11761145; doi:10.1371/journal.pgen.1011548)

**Supplemental Figure 8**: Volcano plots represent log2 fold change by -log10 of eFDR, 0.05, for each of the comparisons A) DES vs. DOM, B) DES vs. CDOM, and C) DOM vs. CDOM. DOM = previously dominant males that remain dominant; DES = previously dominant males that socially descend; CDOM = control dominant animals that remain dominant. Each volcano plot is annotated with genes with largest fold changes as well as lines indicating log2 fold change at 0.5 and 0.75. D) The total number of DEGs, top ten genes with the largest fold change, and corresponding top biological processes GO-terms**.**


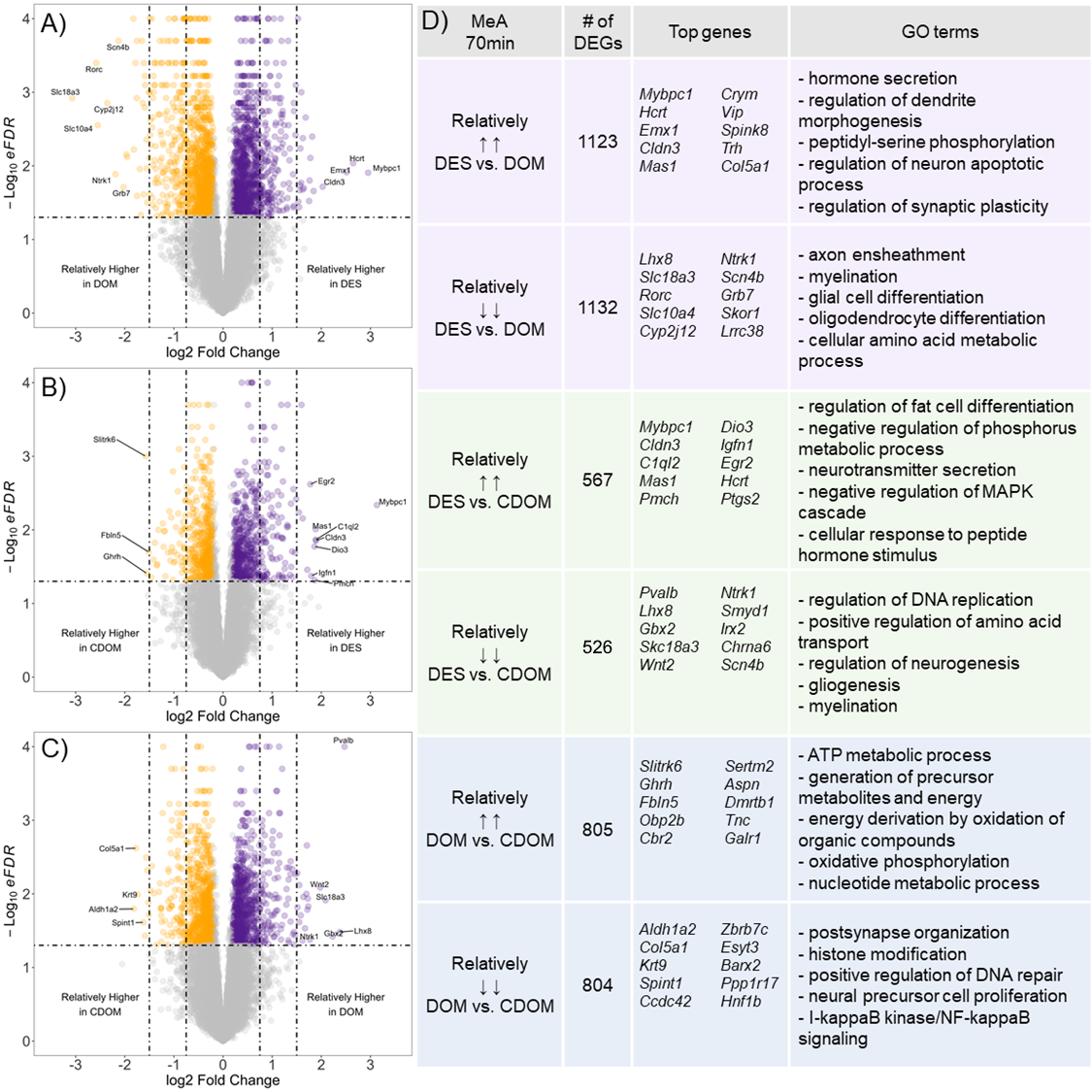

Supplement: S8 Fig — Volcano plots represent log2 fold change by -log10 of eFDR, 0.05, for each of the comparisons A) DES vs. DOM, B) DES vs. CDOM, and C) DOM vs. CDOM. DOM = previously dominant males that remain dominant; DES = previously dominant males that socially descend; CDOM = control dominant animals that remain dominant. Each volcano plot is annotated with genes with largest fold changes as well as lines indicating log2 fold change at 0.5 and 0.75. D) The total number of DEGs, top ten genes with the largest fold change, and corresponding top biological processes GO-terms. (DOCX) [file pgen.1011548.s009.docx]
