# Supplementary material for: Rapid changes in plasma corticosterone and medial amygdala transcriptome profiles during social status change reveal molecular pathways associated with a major life history transition in mouse dominance hierarchies
Source: PLoS Genet. 2025 Jan 13;21(1):e1011548. doi: 10.1371/journal.pgen.1011548 (PMC11761145; doi:10.1371/journal.pgen.1011548)

**Supplemental Figure 9:**  Volcano plots represent log2 fold change by -log10 of eFDR, 0.05, for each of the comparisons A) ASC vs. SUB, B) ASC vs. CSUB, and C) SUB vs. CSUB. SUB = previously subordinate males that remain subordinate; ASC = previously subordinate males that socially ascend; CSUB = control subordinate animals that remain subordinate. Each volcano plot is annotated with genes with largest fold changes as well as lines indicating log2 fold change at 0.5 and 0.75. D) The total number of DEGs, top ten genes with the largest fold change, and corresponding top biological processes GO-terms**.**


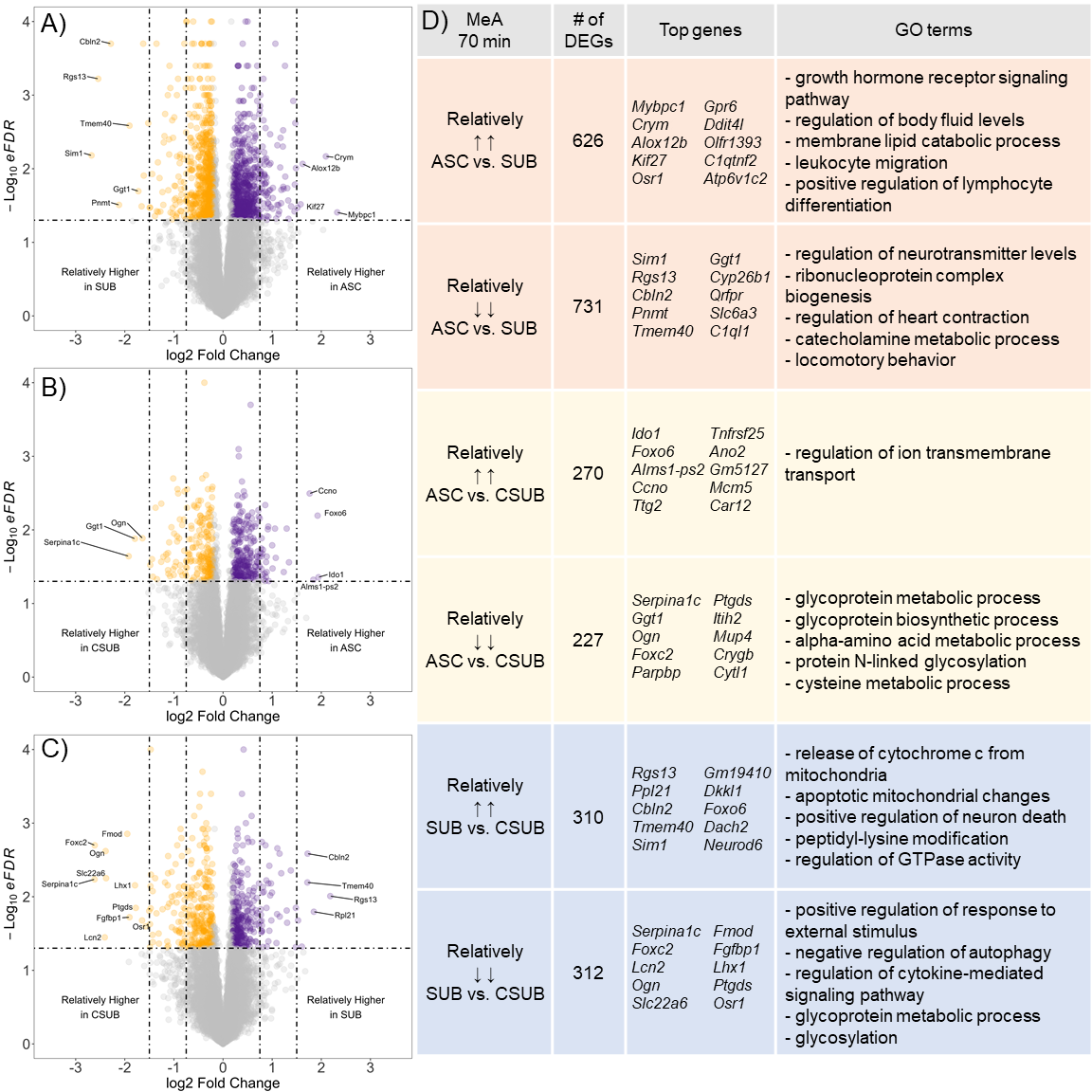

Supplement: S9 Fig — Volcano plots represent log2 fold change by -log10 of eFDR, 0.05, for each of the comparisons A) ASC vs. SUB, B) ASC vs. CSUB, and C) SUB vs. CSUB. SUB = previously subordinate males that remain subordinate; ASC = previously subordinate males that socially ascend; CSUB = control subordinate animals that remain subordinate. Each volcano plot is annotated with genes with largest fold changes as well as lines indicating log2 fold change at 0.5 and 0.75. D) The total number of DEGs, top ten genes with the largest fold change, and corresponding top biological processes GO-terms. (DOCX) [file pgen.1011548.s010.docx]
