# Supplementary material for: Rapid changes in plasma corticosterone and medial amygdala transcriptome profiles during social status change reveal molecular pathways associated with a major life history transition in mouse dominance hierarchies
Source: PLoS Genet. 2025 Jan 13;21(1):e1011548. doi: 10.1371/journal.pgen.1011548 (PMC11761145; doi:10.1371/journal.pgen.1011548)
Supplement: S10 Fig — DEGs met criteria if the absolute values of log2 fold change were greater than 20% at the empirical false discovery rate (eFDR) of 5%. (DOCX) [file pgen.1011548.s011.docx]

**Supplemental Figure 10:** Volcano plots showing log2 fold change and significance (eFDR) for genes in the primary response gene set curated from Tyssowski et al., 2018 [1]. DEGs met criteria if the absolute values of log2 fold change were greater than 15% at the empirical false discovery rate (eFDR) of 5%.


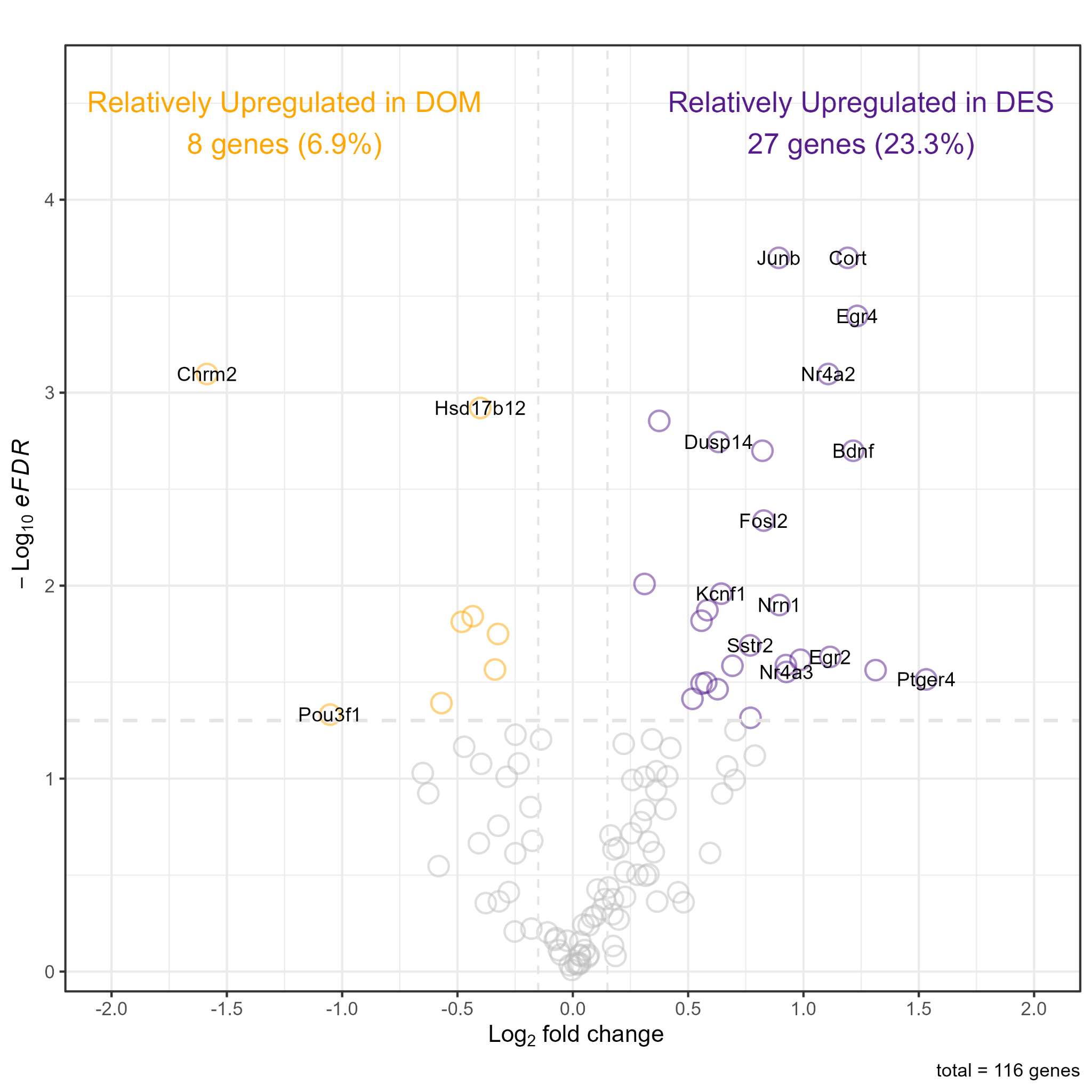

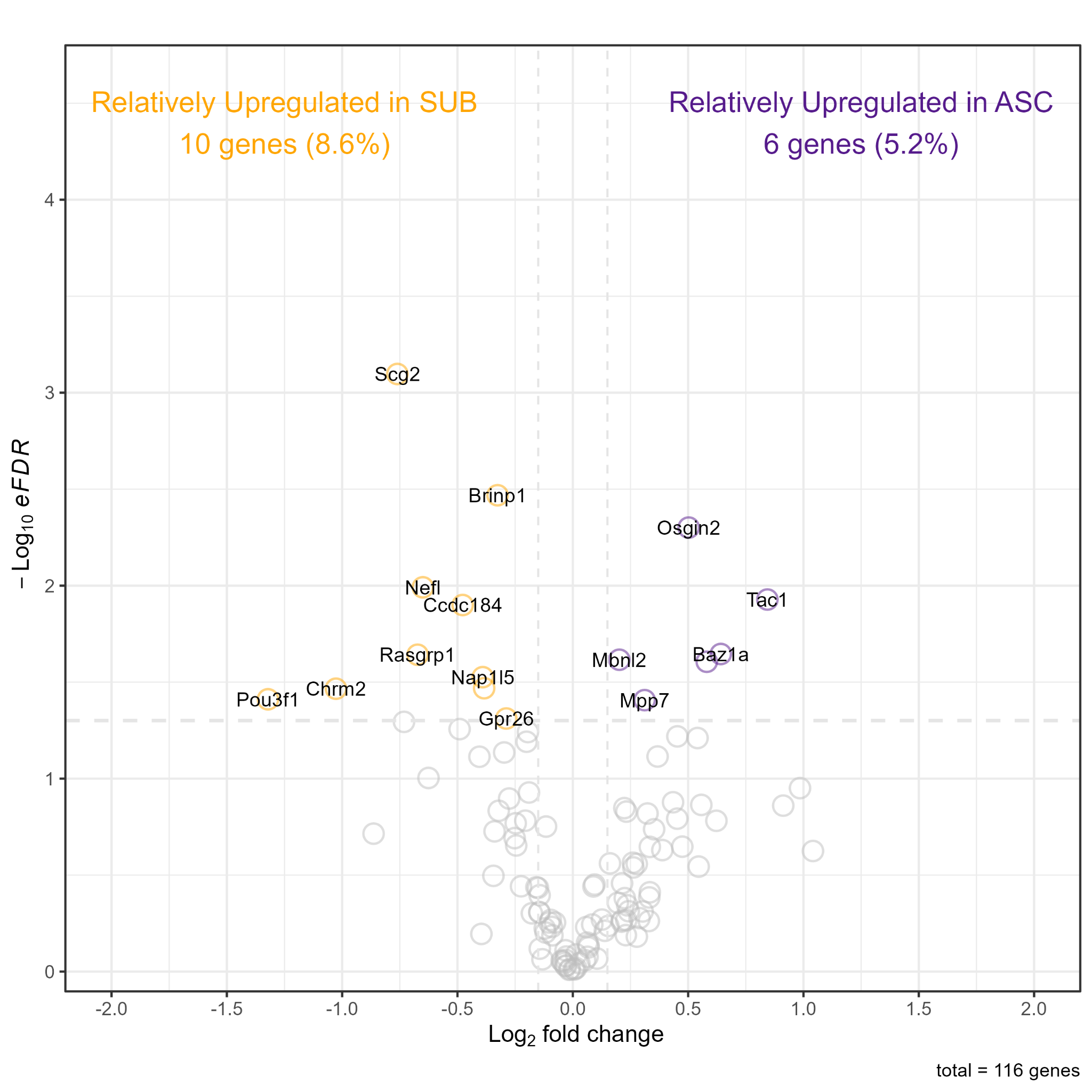


A) Previously Dominant B) Previously Subordinate

**References**

1. Tyssowski KM, DeStefino NR, Cho J-H, Dunn CJ, Poston RG, Carty CE, et al. Different Neuronal Activity Patterns Induce Different Gene Expression Programs. Neuron. 2018;98: 530-546.e11. doi:10.1016/j.neuron.2018.04.001
