# Supplementary material for: Rapid changes in plasma corticosterone and medial amygdala transcriptome profiles during social status change reveal molecular pathways associated with a major life history transition in mouse dominance hierarchies
Source: PLoS Genet. 2025 Jan 13;21(1):e1011548. doi: 10.1371/journal.pgen.1011548 (PMC11761145; doi:10.1371/journal.pgen.1011548)
Supplement: S12 Fig — A) Phred Scores for all sequencing files used in MeA transcriptome analysis. All samples passed sequencing quality checks having a Phred Score > 30. B) Mean input and mapped reads across each condition. (DOCX) [file pgen.1011548.s013.docx]

**Supplemental Figure 12:** A) Phred Scores for all sequencing files used in MeA transcriptome analysis. All samples passed sequencing quality checks having a Phred Score > 30. B) Mean input and mapped reads across each condition.

**
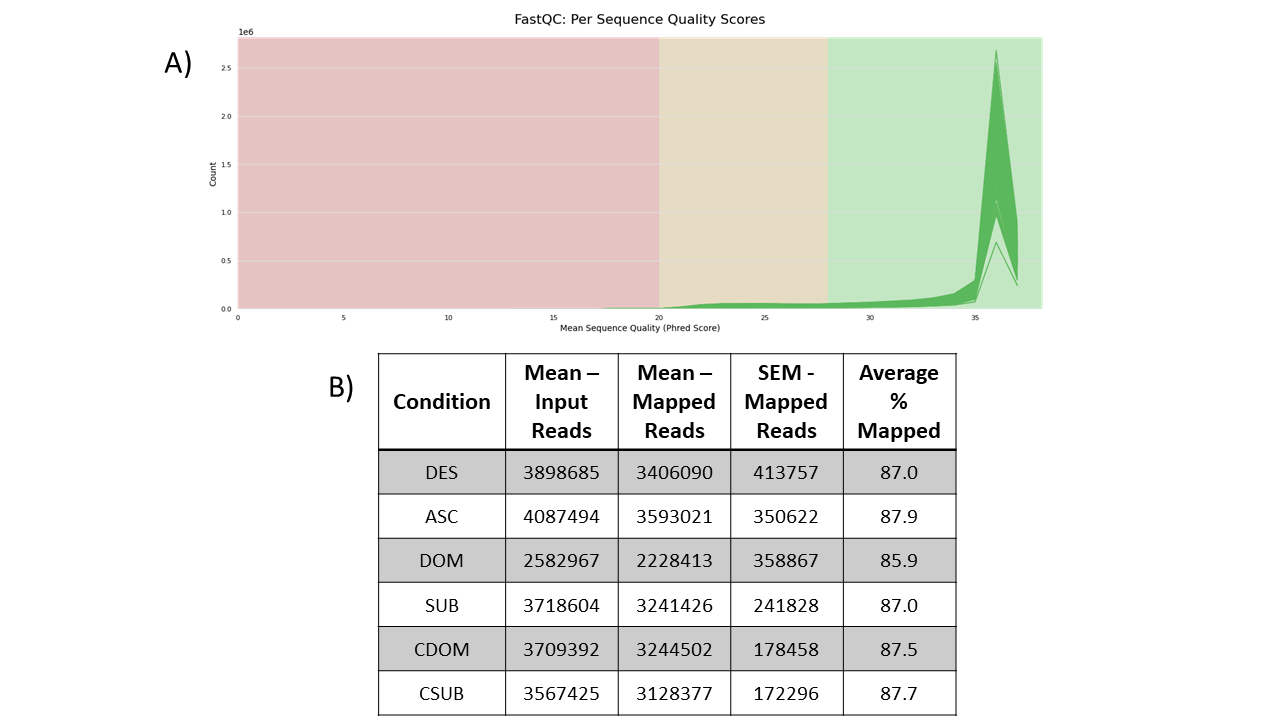
**
