# Supplementary material for: Rapid changes in plasma corticosterone and medial amygdala transcriptome profiles during social status change reveal molecular pathways associated with a major life history transition in mouse dominance hierarchies
Source: PLoS Genet. 2025 Jan 13;21(1):e1011548. doi: 10.1371/journal.pgen.1011548 (PMC11761145; doi:10.1371/journal.pgen.1011548)

**Supplemental Figure 13:** WGCNA Soft threshold plots (I) Dendrograms (II) Module correlation heatmaps (III) for all social conditions (A) and reorganized conditions (B).
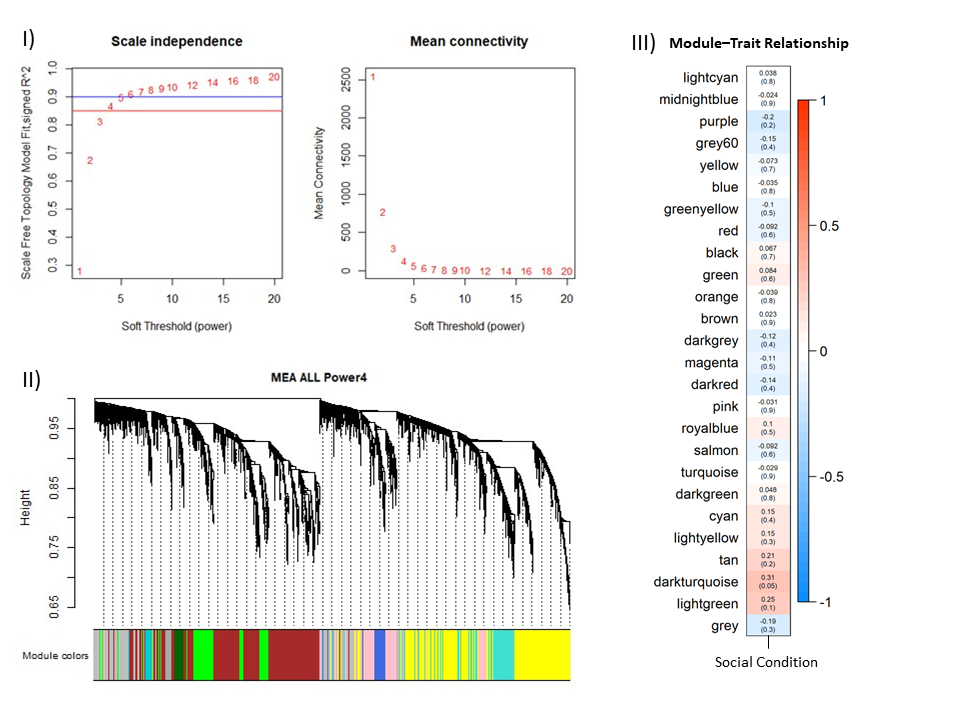


A)

B)


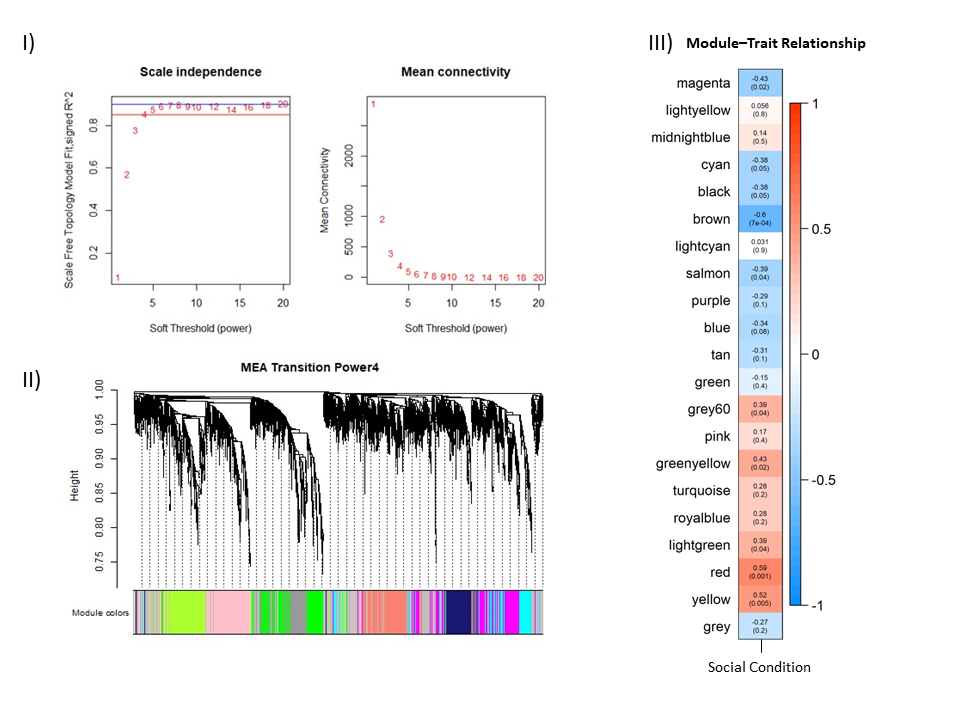

Supplement: S13 Fig — WGCNA Soft threshold plots (I) Dendrograms (II) Module correlation heatmaps (III) for all social conditions (A) and reorganized conditions (B). (DOCX) [file pgen.1011548.s014.docx]
