# Supplementary material for: Rapid changes in plasma corticosterone and medial amygdala transcriptome profiles during social status change reveal molecular pathways associated with a major life history transition in mouse dominance hierarchies
Source: PLoS Genet. 2025 Jan 13;21(1):e1011548. doi: 10.1371/journal.pgen.1011548 (PMC11761145; doi:10.1371/journal.pgen.1011548)

**Supplemental Table 1** –Overlap in DEGs between reorganized and control males. There was a significant consistency in the directionality of expression of DEGs between previously dominant males that maintained rank (DOM) or descended in rank (DES) when each was compared to control dominants (CDOM) (Chi-Squared test: χ2=155.65, p<0.001, Φ=0.87), as well as between previously subordinate males that maintained rank (SUB) or ascended in rank (ASC) when each was compared to control subordinates (CSUB) (Chi-Squared test: χ2=65.02, p<0.001, Φ=1.00). One gene, Mt3 (Metallothionein-3) was upregulated in all comparisons (DES vs cDOM log2FC=0.51, p=0.009; DOM vs cDOM log2FC=0.47, p=0.017; ASC vs cSUB log2FC=0.43, p=0.047; SUB vs cSUB log2FC=0.49, p=0.015).


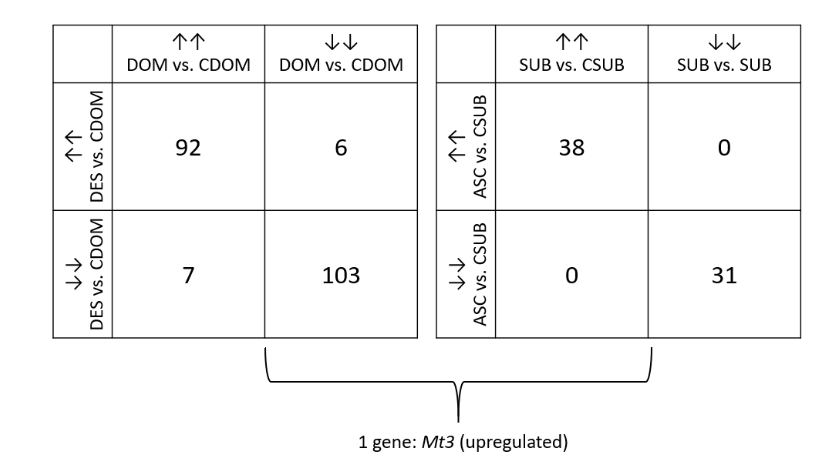

Supplement: S1 Table — There was a significant consistency in the directionality of expression of DEGs between previously dominant males that maintained rank (DOM) or descended in rank (DES) when each was compared to control dominants (CDOM) (Chi-Squared test: χ2 = 155.65, p<0.001, Φ = 0.87), as well as between previously subordinate males that maintained rank (SUB) or ascended in rank (ASC) when each was compared to control subordinates (CSUB) (Chi-Squared test: χ2 = 65.02, p<0.001, Φ = 1.00). One gene, Mt3 (Metallothionein-3) was upregulated in all comparisons (DES vs cDOM log2FC = 0.51, p = 0.009; DOM vs cDOM log2FC = 0.47, p = 0.017; ASC vs cSUB log2FC = 0.43, p = 0.047; SUB vs cSUB log2FC = 0.49, p = 0.015). (DOCX) [file pgen.1011548.s015.docx]
