# Supplementary material for: Rapid changes in plasma corticosterone and medial amygdala transcriptome profiles during social status change reveal molecular pathways associated with a major life history transition in mouse dominance hierarchies
Source: PLoS Genet. 2025 Jan 13;21(1):e1011548. doi: 10.1371/journal.pgen.1011548 (PMC11761145; doi:10.1371/journal.pgen.1011548)
Supplement: S2 Table — Each gene is significantly differentially expressed in both DES vs DOM (descending animals vs animals who maintained dominant status) and ASC vs SUB (ascending animals vs animals who maintained subordinate status). (DOCX) [file pgen.1011548.s016.docx]

**Supplemental Table 2** - Social Transition genes. Each gene is significantly differentially expressed in both DES vs DOM (descending animals vs animals who maintained dominant status) and ASC vs SUB (ascending animals vs animals who maintained subordinate status).

| **symbol** | **DES_logFC** | **DES_pvalue** | **ASC_logFC** | **ASC_pvalue** |
| --- | --- | --- | --- | --- |
| A830018L16Rik | 0.576914 | 4.00E-04 | 0.329162 | 0.0464 |
| Abcf1 | -0.24094 | 0.0014 | -0.23414 | 0.0024 |
| Ache | -0.50638 | 0.0146 | -0.5216 | 0.0408 |
| Actn1 | 0.512624 | 0.001 | 0.463429 | 0.019 |
| Acvr1c | 1.216223 | 0.005 | 0.776506 | 0.021 |
| Adamts15 | 0.493878 | 0.0284 | 0.359582 | 0.0328 |
| Adcy2 | -0.67237 | 0.0128 | -0.57349 | 0.0342 |
| Adnp2 | 0.364177 | 0.0226 | 0.295345 | 0.0026 |
| Ak6 | -0.22997 | 0.0426 | -0.25215 | 0.0282 |
| Akap12 | -0.54412 | 0.0152 | -0.64061 | 0.0212 |
| Akr1b10 | -0.70806 | 0.0112 | -0.49523 | 0.0192 |
| Alkbh1 | 0.429255 | 0.0336 | 0.269539 | 0.019 |
| Aloxe3 | 0.801568 | 0.0066 | 0.698718 | 0.0094 |
| Ank1 | -0.65354 | 0.006 | -0.54263 | 0.0216 |
| Ano3 | 0.657585 | 0.0024 | 0.589725 | 0.0412 |
| Aplp2 | -0.25217 | 0.0022 | -0.26624 | 0.0488 |
| Arhgap33 | 0.41112 | 0.0068 | 0.645786 | 0.0076 |
| Arnt2 | -0.29393 | 0.019 | -0.22513 | 0.0402 |
| Atf7 | 0.241682 | 0.0344 | 0.252455 | 0.0208 |
| Atp1b1 | -0.21439 | 0.0166 | -0.28514 | 0.0316 |
| Atp2c1 | 0.3681 | 0.0062 | 0.303319 | 0.0022 |
| Atp6v1c2 | 0.90329 | 0.0032 | 1.42903 | 0.0012 |
| Auts2 | 0.595016 | 0.0016 | 0.478779 | 0.0134 |
| Bcor | -0.41408 | 0.0114 | -0.34416 | 0.0082 |
| Bean1 | -0.35088 | 0.0122 | -0.38788 | 0.0048 |
| Bmper | -0.73108 | 0.0484 | -0.87735 | 0.007 |
| C2cd2l | 0.51791 | 4.00E-04 | 0.400995 | 0.039 |
| Car12 | 1.59098 | 0.017 | 1.217787 | 0.005 |
| Carf | 0.286358 | 0.0218 | 0.292225 | 0.011 |
| Cbln2 | -1.29416 | 0.0306 | -2.28456 | 2.00E-04 |
| Ccdc116 | 0.535357 | 0.0432 | 0.491627 | 0.029 |
| Ccdc149 | -0.24663 | 0.0422 | -0.3631 | 0.0038 |
| Ccdc163 | -0.47023 | 0.0256 | -0.48242 | 0.0422 |
| Ccdc186 | -0.31574 | 0.0092 | -0.35766 | 0.001 |
| Ccdc28b | -0.42506 | 0.0066 | -0.31516 | 0.038 |
| Cdh13 | 0.347072 | 0.0044 | 0.390885 | 0.0044 |
| Cep290 | -0.2941 | 0.014 | -0.28524 | 0.012 |
| Cep43 | -0.40281 | 4.00E-04 | -0.41888 | 0.0134 |
| Cert1 | -0.46004 | 2.00E-04 | -0.30273 | 0.0206 |
| Cgrrf1 | -0.40165 | 0.004 | -0.29726 | 0.0128 |
| Chga | -0.33121 | 0.0452 | -0.46375 | 0.0234 |
| Chrm2 | -1.58609 | 8.00E-04 | -1.02681 | 0.0342 |
| Chst11 | 0.334468 | 0.0332 | 0.49657 | 0.01 |
| Cinp | 0.26354 | 0.0388 | 0.277402 | 0.0348 |
| Cisd3 | -0.38816 | 0.0118 | -0.38069 | 0.0212 |
| Coasy | -0.33388 | 0.0038 | -0.36104 | 0.0204 |
| Col11a1 | -1.06369 | 0.009 | -0.74663 | 0.036 |
| Col23a1 | 0.953247 | 2.00E-04 | 0.82572 | 0.0296 |
| Col6a1 | 0.617155 | 0.0244 | 1.232929 | 0.0188 |
| Cpd | 0.434778 | 0.0102 | 0.353497 | 0.0278 |
| Cplane2 | 0.463229 | 0.037 | 0.484374 | 0.0342 |
| Crym | 1.814335 | 0.004 | 2.09364 | 0.0068 |
| Ctps2 | -0.25534 | 0.0088 | -0.27367 | 0.0036 |
| Cxadr | 0.446209 | 0.0284 | 0.27315 | 0.0254 |
| Cybrd1 | 0.546543 | 0.0458 | 0.580657 | 0.0264 |
| Cyp26b1 | -1.43612 | 0.0274 | -1.65781 | 0.0324 |
| Dapk1 | 0.553517 | 0.005 | 0.483142 | 4.00E-04 |
| Dclk3 | 0.673757 | 0.0414 | 0.653459 | 0.0386 |
| Ddn | 1.178228 | 0.002 | 1.105207 | 0.0048 |
| Ddx1 | -0.24543 | 0.0172 | -0.2106 | 0.0262 |
| Dennd1a | 0.319858 | 0.0148 | 0.254854 | 0.004 |
| Dennd1b | 0.454137 | 0.0108 | 0.353434 | 0.0324 |
| Doc2b | 0.651758 | 0.0092 | 0.728428 | 0.0134 |
| DPCD | -0.24077 | 0.018 | -0.27905 | 0.0096 |
| Dpysl3 | -0.35217 | 0.008 | -0.43594 | 0.0288 |
| Efcab2 | -0.40986 | 0.0022 | -0.4175 | 0.0144 |
| Elavl2 | -0.4304 | 0.0228 | -0.64386 | 0.0134 |
| Endod1 | -0.55953 | 0.0016 | -0.35774 | 0.0332 |
| Erc2 | 0.368751 | 0.0444 | 0.45501 | 0.014 |
| Esrrb | -1.37009 | 0.0374 | -1.38596 | 0.044 |
| Fam124a | 0.644842 | 0.0024 | 0.500069 | 0.0198 |
| Fam126a | 0.362332 | 0.0126 | 0.529068 | 0.0034 |
| Fam184b | 0.558935 | 0.004 | 0.583888 | 0.0206 |
| Fam187b | 0.591803 | 0.0238 | 0.521903 | 0.0166 |
| Fam227a | 0.361337 | 0.04 | 0.389217 | 0.0164 |
| Fat4 | 0.978463 | 0.0286 | 0.881973 | 0.0154 |
| Fcf1 | -0.42539 | 0.0274 | -0.32471 | 0.0392 |
| Fhod3 | -0.54206 | 0.0396 | -0.67316 | 0.0038 |
| Fibin | 0.919316 | 0.0048 | 0.901467 | 0.0346 |
| Fignl2 | -0.95528 | 2.00E-04 | -0.74508 | 0.048 |
| Flrt2 | -0.46333 | 0.0104 | -0.28006 | 0.035 |
| Flywch1 | -0.32962 | 0.0034 | -0.28725 | 0.0268 |
| Fundc2 | -0.44095 | 0.0018 | -0.5013 | 0.0014 |
| Fuom | -0.31081 | 0.0388 | -0.29351 | 0.037 |
| Fxyd5 | 0.460113 | 0.009 | 0.531923 | 0.0306 |
| Gda | 0.714907 | 0.0044 | 0.696404 | 0.0386 |
| Glrx3 | -0.25967 | 0.0226 | -0.24639 | 0.031 |
| Gm5176 | 0.803507 | 0.019 | 0.417904 | 0.0218 |
| Gnas | -0.20436 | 0.0092 | -0.34424 | 0.004 |
| Gpc4 | 0.787776 | 0.0384 | 0.887491 | 0.0132 |
| Gpr153 | -0.66294 | 0.0118 | -0.50162 | 0.0492 |
| Gria3 | 0.351539 | 0.0342 | 0.42329 | 0.0256 |
| Grid1 | 0.443613 | 0 | 0.254416 | 0.0046 |
| Grin2b | 0.522996 | 0.0134 | 0.428451 | 0.0384 |
| Gtpbp2 | 0.328593 | 0.0282 | 0.381475 | 8.00E-04 |
| H2-DMa | 0.658192 | 0.0016 | 0.596477 | 0.0254 |
| Haus3 | -0.58708 | 0.0024 | -0.4221 | 0.0024 |
| Heatr3 | 0.284168 | 0.0426 | 0.270441 | 0.029 |
| Hipk2 | -0.48698 | 0.0022 | -0.25815 | 0.0224 |
| Hivep1 | -0.60082 | 0.0038 | -0.33175 | 0.01 |
| Hrk | 1.067184 | 0.0028 | 0.913428 | 0.029 |
| Hsp90aa1 | -0.2872 | 0.024 | -0.32219 | 0.0012 |
| Hspa4l | -0.31339 | 8.00E-04 | -0.34043 | 0.0274 |
| Htr4 | 1.272175 | 0.004 | 1.261285 | 4.00E-04 |
| Ifitm10 | -0.597 | 0.0112 | -0.56761 | 0.0292 |
| Iglon5 | -0.76236 | 0.0158 | -0.47027 | 0.021 |
| Ikzf4 | 0.446295 | 6.00E-04 | 0.326641 | 0.0456 |
| Il17rd | 0.583291 | 0.0108 | 0.497763 | 0.032 |
| Ints12 | -0.44733 | 0.043 | -0.44184 | 0.0054 |
| Itpk1 | -0.5648 | 6.00E-04 | -0.58345 | 0.0018 |
| Itpka | 1.058407 | 0.0078 | 0.970381 | 0.033 |
| Jph4 | 0.362694 | 0.0038 | 0.363882 | 0.0304 |
| Kcng1 | 0.611461 | 0.0394 | 0.487877 | 0.0388 |
| Kcnh4 | 1.359431 | 0.0206 | 1.334375 | 0.048 |
| Kcnj4 | 0.727073 | 0.0176 | 0.608933 | 0.035 |
| Kcns1 | 1.444563 | 0.0018 | 0.860838 | 0.0162 |
| Kif21a | -0.26174 | 0.0024 | -0.21934 | 0.0452 |
| Kin | -0.28857 | 0.0386 | -0.33757 | 0.0262 |
| Klhl1 | -0.98688 | 0.0126 | -1.02625 | 0.0406 |
| Klhl4 | -0.7249 | 0.0224 | -0.69851 | 0.048 |
| Lamb1 | 0.777684 | 0.0084 | 0.528155 | 0.021 |
| Laptm4b | -0.36561 | 0.011 | -0.65439 | 0.007 |
| Lcorl | 0.624974 | 0.0174 | 0.539802 | 0.0372 |
| Lhfpl3 | -0.75376 | 0.0044 | -0.40404 | 0.026 |
| Limk1 | -0.52605 | 0.0158 | -0.52866 | 0.0234 |
| Lin37 | -0.23619 | 0.0402 | -0.28398 | 0.0192 |
| Lingo3 | 0.375145 | 0.032 | 0.626091 | 0.01 |
| Lmo7 | 0.677537 | 0.0174 | 0.805429 | 0.005 |
| Lpl | 0.673022 | 0.0234 | 1.065266 | 2.00E-04 |
| Lrrc4c | 0.23567 | 0.0088 | 0.203906 | 0.0404 |
| Luzp1 | -0.5871 | 0.0012 | -0.35687 | 0.0466 |
| Magoh | -0.33315 | 0.0362 | -0.20552 | 0.0324 |
| Map7d2 | -0.25013 | 0.026 | -0.31431 | 0.0496 |
| Mast3 | 0.530595 | 0.0012 | 0.446671 | 0.0126 |
| Mchr1 | 0.950051 | 6.00E-04 | 0.500846 | 0.0226 |
| Mr1 | 0.628206 | 0.0434 | 0.67835 | 0.0066 |
| Mrps18a | -0.26762 | 0.038 | -0.38989 | 0.0112 |
| Mrtfa | 0.292921 | 0.0332 | 0.344639 | 0.0254 |
| Mtmr12 | 0.653097 | 0.0138 | 0.503465 | 0.039 |
| Mxd3 | 1.481602 | 8.00E-04 | 0.95914 | 0.0034 |
| Mybpc1 | 2.952795 | 0.0124 | 2.324035 | 0.0392 |
| Mzf1 | 0.381506 | 0.0266 | 0.593283 | 0.0026 |
| Naa40 | 0.359234 | 0.0114 | 0.280414 | 0.0082 |
| Nap1l3 | -0.4735 | 2.00E-04 | -0.38543 | 0.0024 |
| Nap1l5 | -0.43397 | 0.0144 | -0.39062 | 0.0298 |
| Nars | -0.22661 | 0.0402 | -0.31341 | 0.0132 |
| Nedd4l | 0.290788 | 0.0376 | 0.237881 | 0.0462 |
| Nefl | -0.32416 | 0.0178 | -0.65017 | 0.0102 |
| Nefm | -0.72906 | 0.009 | -0.98883 | 0.017 |
| Nek10 | 0.67313 | 0.0022 | 0.6255 | 0.0042 |
| Neurl1a | 0.357858 | 0.0362 | 0.362629 | 0.0384 |
| Neurl1b | 0.63852 | 0.0038 | 0.39567 | 0.0278 |
| Nhsl2 | 0.336584 | 0.0024 | 0.252126 | 0.0276 |
| Nkrf | -0.50713 | 0.0018 | -0.35424 | 0.0212 |
| Nphp4 | 0.356758 | 0.029 | 0.41922 | 0.0134 |
| Nrbp2 | 0.333131 | 0.008 | 0.277029 | 0.0132 |
| Nrip1 | 0.473659 | 0.0326 | 0.552179 | 0.001 |
| Nsmf | 0.404612 | 0.042 | 0.468741 | 0.0266 |
| Olfr1247 | 0.399742 | 0.0194 | 0.393881 | 0.0442 |
| Olfr393 | 0.634442 | 0.0224 | 0.523329 | 0.0446 |
| Olfr801 | 1.136483 | 0.0012 | 0.736895 | 0.0018 |
| Olfr806 | 1.077083 | 4.00E-04 | 0.889375 | 0.008 |
| Otulinl | 0.788781 | 0.009 | 0.557488 | 0.0128 |
| P3h3 | 0.474064 | 0.0036 | 0.423678 | 0.0368 |
| Paqr9 | 0.367383 | 0.001 | 0.339252 | 0.01 |
| Pcdh10 | -0.37534 | 0.0452 | -0.56917 | 0.0054 |
| Pcdh17 | 0.614655 | 0.0162 | 0.623496 | 0.014 |
| Pced1b | 0.383474 | 0.0322 | 0.463611 | 0.0084 |
| Pde4d | -0.41217 | 0.0064 | -0.43293 | 0.0124 |
| Pde7a | 0.298642 | 0.0388 | 0.227181 | 0.0406 |
| Peli1 | 0.453161 | 0.0014 | 0.410082 | 0.0012 |
| Pfdn2 | -0.30176 | 0.0374 | -0.30249 | 0.032 |
| Pfdn4 | -0.22671 | 0.0182 | -0.3496 | 0.0032 |
| Phlda3 | -1.03098 | 0.0014 | -0.62 | 0.0042 |
| Pitpnm2 | 0.464737 | 0.0334 | 0.632129 | 0.007 |
| Plcb4 | -0.54672 | 0.0092 | -0.47668 | 0.0442 |
| Plekhg5 | 0.410661 | 0.0296 | 0.401116 | 0.0066 |
| Plekho1 | -0.38049 | 0.0292 | -0.40098 | 0.0134 |
| Plppr4 | 0.442826 | 0.0206 | 0.43545 | 0.0362 |
| Pou3f1 | -1.05217 | 0.0466 | -1.32156 | 0.0388 |
| Ppl | 0.938337 | 0.033 | 0.739253 | 0.0424 |
| Ppp3ca | 0.415196 | 6.00E-04 | 0.442584 | 0.0124 |
| Prdm10 | 0.361716 | 0.0064 | 0.256656 | 0.0462 |
| Prdx4 | -0.41645 | 0.0346 | -0.37581 | 0.0354 |
| Prickle1 | -0.368 | 0.0154 | -0.33442 | 0.0058 |
| Prkce | 0.418845 | 0.0076 | 0.335102 | 0.0356 |
| Ptpn5 | 0.261262 | 0.0166 | 0.516394 | 0.004 |
| Ptpre | 0.490026 | 0.0024 | 0.41017 | 0.004 |
| Rab40b | 0.731293 | 0.011 | 0.670945 | 0.0218 |
| Rad21 | -0.29776 | 0.002 | -0.24244 | 0.005 |
| Rai2 | -0.75914 | 0.002 | -0.78846 | 2.00E-04 |
| Rasal2 | 0.353864 | 0.01 | 0.213206 | 0.023 |
| Rdx | -0.4619 | 0 | -0.30822 | 0.0138 |
| Rfx3 | 0.890071 | 0.005 | 0.624098 | 0.0346 |
| Rgs3 | -0.61217 | 0.0058 | -0.36475 | 0.0428 |
| Rhbdl3 | 0.512565 | 0.024 | 0.35822 | 0.049 |
| Rin1 | 0.785969 | 0.0258 | 0.849802 | 0.0158 |
| Rit2 | -0.33272 | 0.0114 | -0.3939 | 0.0236 |
| Rp9 | -0.33514 | 0.0076 | -0.21521 | 0.0154 |
| Rreb1 | 1.014476 | 0.0012 | 0.922614 | 0.0448 |
| Rwdd2b | 0.564129 | 0.0302 | 0.383454 | 0.0058 |
| Scrn1 | -0.31135 | 0.0088 | -0.30854 | 0.0346 |
| Sema3c | -0.84787 | 0.0018 | -0.79478 | 0.0152 |
| Sema6d | -0.78823 | 6.00E-04 | -0.53232 | 0.01 |
| Septin6 | -0.54138 | 0.012 | -0.60273 | 8.00E-04 |
| Sfxn2 | 0.220636 | 0.0294 | 0.335181 | 0.0186 |
| Shisa7 | 0.477031 | 0.0024 | 0.416816 | 0.0376 |
| Shox2 | -1.25789 | 0.0474 | -1.49443 | 0.0338 |
| Ski | 0.309764 | 0.0212 | 0.41119 | 0.0156 |
| Slc16a2 | 0.75067 | 0.0012 | 0.65579 | 0.0362 |
| Slc25a37 | 0.503164 | 0.0014 | 0.267756 | 0.0398 |
| Slc6a3 | -1.11624 | 0.0426 | -1.61349 | 0.0124 |
| Smad3 | 0.74133 | 0.0142 | 0.722663 | 0.0272 |
| Smpd3 | 0.349754 | 0.0174 | 0.433185 | 0.032 |
| Smpdl3b | 1.645859 | 0.02 | 0.889549 | 0.0438 |
| Sowaha | 0.875618 | 2.00E-04 | 0.851554 | 0.0074 |
| Sox12 | 0.245088 | 0.011 | 0.253601 | 0.027 |
| Sox4 | -0.37394 | 0.0152 | -0.27275 | 0.0138 |
| Sphk2 | -0.35872 | 0.024 | -0.34146 | 0.0024 |
| Stk32b | -0.7038 | 0.0306 | -0.96931 | 0.0308 |
| Stk39 | -0.53515 | 0.0016 | -0.47268 | 0.0094 |
| Stxbp2 | 0.355786 | 0.0014 | 0.377291 | 0.0152 |
| Styx | 0.253866 | 0.0384 | 0.370963 | 0.0092 |
| Supt16 | -0.39379 | 0.0032 | -0.27883 | 2.00E-04 |
| Sv2a | -0.32726 | 0.001 | -0.3303 | 0.0378 |
| Syt1 | -0.21959 | 0.0144 | -0.35423 | 0.009 |
| Taf7 | -0.3526 | 0.0238 | -0.41964 | 0.0044 |
| Tax1bp1 | -0.2211 | 0.0094 | -0.22313 | 0.0058 |
| Tcof1 | -0.27138 | 0.0444 | -0.26691 | 0.0176 |
| Tenm2 | 0.437471 | 0.0148 | 0.482142 | 0.0028 |
| Tesc | 0.52331 | 0.0284 | 0.837322 | 0.006 |
| Th | -1.25823 | 0.0038 | -0.88291 | 0.0084 |
| Tlk2 | -0.368 | 4.00E-04 | -0.26717 | 0.0214 |
| Tmem65 | -0.71275 | 0.02 | -1.01694 | 0.0038 |
| Tmf1 | -0.22637 | 0.033 | -0.37024 | 0.0016 |
| Tonsl | -0.72035 | 0.0014 | -0.49261 | 0.0486 |
| Trabd2b | 1.341581 | 0.0172 | 1.182776 | 0.0294 |
| Traip | 0.594975 | 0.0196 | 0.864589 | 0.0204 |
| Trhr | 0.896836 | 0.0248 | 0.960687 | 0.0194 |
| Trim12a | 0.339964 | 0.041 | 0.382985 | 0.0082 |
| Trpc1 | 0.308223 | 0.034 | 0.358899 | 0.003 |
| Tspan17 | -0.64869 | 0.0136 | -0.6565 | 0.0038 |
| Tspoap1 | 0.235766 | 0.0498 | 0.329868 | 0.0152 |
| Ttc12 | -0.64165 | 0.005 | -0.61505 | 0.0302 |
| Uchl5 | -0.22014 | 0.0214 | -0.29699 | 0.0094 |
| Unc13a | 0.445289 | 0.007 | 0.329002 | 0.0176 |
| Usp6nl | -0.35053 | 0.006 | -0.24788 | 0.0228 |
| Vat1l | -0.55571 | 0.0416 | -0.40414 | 0.0404 |
| Vmn1r181 | 0.594437 | 0.022 | 0.446644 | 0.0258 |
| Vmn1r213 | 0.278584 | 0.0226 | 0.375649 | 0.0354 |
| Vps36 | -0.21551 | 0.0152 | -0.28007 | 0.028 |
| Vwc2 | -0.39979 | 0.0272 | -0.58621 | 0.0274 |
| Vwc2l | -0.78563 | 0.0038 | -0.57565 | 0.0422 |
| Wnk4 | 0.777488 | 0.026 | 1.127554 | 0.0416 |
| Zbtb1 | 0.317111 | 0.0204 | 0.25438 | 0.014 |
| Zbtb38 | -0.22866 | 0.005 | -0.24757 | 0 |
| Zcchc2 | -0.30881 | 0.049 | -0.29425 | 0.0352 |
| Zdhhc23 | 0.759351 | 0.0242 | 0.693417 | 0.0056 |
| Zdhhc5 | -0.23269 | 0.0478 | -0.29157 | 0.0242 |
| Zfp101 | -0.45087 | 0.0294 | -0.66863 | 0.0314 |
| Zfp14 | -0.56339 | 0.0106 | -0.32818 | 0.0366 |
| Zfp512b | 0.224156 | 0.0364 | 0.263564 | 0.0192 |
| Zic5 | 1.593859 | 0.001 | 1.225841 | 0.0176 |
